# Comparisons of resistance of CF and Non-CF pathogens to Hydrogen Peroxide and Hypochlorous Acid Oxidants *In Vitro*

**DOI:** 10.1186/1471-2180-11-112

**Published:** 2011-05-20

**Authors:** Ryan W Bonvillain, Richard G Painter, Elisa M Ledet, Guoshun Wang

**Affiliations:** 1 Department of Genetics, Louisiana State University Health Sciences Center New Orleans, Louisiana 70112, USA; 2 Department of Microbiology, Louisiana State University Health Sciences Center New Orleans, Louisiana 70112, USA; 3 Department of Medicine, Louisiana State University Health Sciences Center New Orleans, Louisiana 70112, USA; 4 Center for Stem Cell Research and Regenerative Medicine, Tulane University Health Sciences Center, New Orleans, LA 70112

**Keywords:** Hydrogen Peroxide, Hypochlorous Acid, Microbicidal, Cystic Fibrosis, Neutrophils, Oxidants

## Abstract

**Background:**

Cystic fibrosis (CF) lung disease has a unique profile of pathogens predominated by *Pseudomonas aeruginosa *(PsA) and *Staphylococcus aureus *(SA). These microorganisms must overcome host immune defense to colonize the CF lungs. Polymorphonuclear neutrophils are a major component of the host defense against bacterial infection. A crucial microbicidal mechanism is the production of oxidants including hydrogen peroxide (H_2_O_2_) and hypochlorous acid (HOCl) by neutrophils to achieve efficient bacterial killing. To determine to what degrees various CF pathogens resist the oxidants relative to non-CF pathogens, we compared the susceptibility of PsA, SA, *Burkholderia cepacia *(BC), *Klebsiella pneumoniae *(KP), and *Escherichia coli *(EC) to various concentrations of H_2_O_2 _or HOCl, *in vitro*. The comparative oxidant-resistant profiles were established. Oxidant-induced damage to ATP production and cell membrane integrity of the microbes were quantitatively assessed. Correlation of membrane permeability and ATP levels with bacterial viability was statistically evaluated.

**Results:**

PsA was relatively resistant to both H_2_O_2 _(LD_50 _= 1.5 mM) and HOCl (LD_50 _= 0.035 mM). SA was susceptible to H_2_O_2 _(LD_50 _= 0.1 mM) but resistant to HOCl (LD_50 _= 0.035 mM). Interestingly, KP was extremely resistant to high doses of H_2_O_2 _(LD_50 _= 2.5-5.0 mM) but was very sensitive to low doses of HOCl (LD_50 _= 0.015 mM). BC was intermediate to resist both oxidants: H_2_O_2 _(LD_50 _= 0.3-0.4 mM) and HOCl (LD_50 _= 0.025 mM). EC displayed the least resistance to H_2_O_2 _(LD_50 _= 0.2-0.3 mM) and HOCl (LD_50 _= 0.015 mM). The identified profile of H_2_O_2_-resistance was KP > PsA > BC > EC > SA and the profile of HOCl-resistance PsA > SA > BC > EC > KP. Moreover, both oxidants affected ATP production and membrane integrity of the cells. However, the effects varied among the tested organisms and, the oxidant-mediated damage correlated differentially with the bacterial viability.

**Conclusions:**

The order of HOCl-resistance identified herein best fits the clinical profile of CF infections. Even though oxidants are able to disrupt ATP production and cell membrane integrity, the degrees of damage vary among the organisms and correlate differentially with their viability.

## Background

Cystic fibrosis (CF) is the most common fatal genetic disease in Caucasians and is caused by mutations of the CF transmembrane conductance regulator (CFTR), a cAMP-stimulated chloride (Cl^-^) channel [1]. The most devastating anomaly of CF is the lung disease which is characterized by chronic bacterial infection, abnormal airway inflammation, extensive neutrophil infiltration and small airway obstruction [2,3]. CF lung infection has a unique pathogen profile which is distinct from other lung infections. *Pseudomonas aeruginosa, Staphylococcus aureus, Haemophilus influenzae*, *Stenotrophomonas maltophilia, Achromobacter xylosoxidans, Burkholderia cepacia *are the most prevalent, among which *P. aeruginosa *predominates [4-6]. Strikingly, all the CF organisms except *S. aureus *are opportunistic pathogens, which do not cause infections in healthy hosts [6]. It is not fully understood why CF patients are particularly susceptible to these organisms and how the organisms manage to escape the host defense at the early infection stage when there is little antibiotic selection and environmental pressure. Apparently, it is the early microbe-host interaction that determines the early pathogen colonization and subsequently persistent infection in CF lungs.

The first line of host defense against invading bacteria is the recruitment of polymorphonuclear neutrophils (PMNs) to sites of infection. Normally, PMNs effectively contain the microbes by phagocytosis and then mount multi-tiered chemical attacks with pre-fabricated and *de novo-*produced agents to kill the phagocytosed organisms [7-9]. The NADPH oxidase-myeloperoxidase (MPO) system constitutes a major antimicrobial mechanism employed by PMNs to fight infections and accounts for ~90% of the oxygen consumed during the phagocyte respiratory burst [10]. This system generates a number of microbicidal oxidants including superoxide (O_2_^-^), hydrogen peroxide (H_2_O_2_), and hypochlorous acid (HOCl) [11], among which HOCl is most potent. HOCl biosynthesis is catalyzed by MPO by using H_2_O_2_, H^+ ^and Cl^- ^as its substrates. As shown in the reaction , the availability of chloride anion in the neutrophil phagosomes limits the production of HOCl. Consequently, any decreased HOCl production reduces H_2_O_2 _consumption, thus affecting the level of H_2_O_2 _in the organelle. Our recent publications demonstrated that the PMNs from CF patients are deficient in chloride transport to phagosomes and deficient in intraphagosomal chlorination and killing of PsA [12-14], suggesting an altered oxidant production in CF neutrophils. Such a defect in phagocytic innate immunity may preferentially allow certain bacterial strains to evade the compromised host defense.

In the current study, we hypothesized that if the HOCl production abnormality in CF neutrophils plays a major role in the disease pathogenesis, then the HOCl-resistant bacteria should be the most clinically prevalent. To test the hypothesis, we sought to investigate the intrinsic resistance of CF and non-CF organisms to H_2_O_2 _and HOCl in a cell-free system. Responses of PsA, SA, BC, KP and EC to the chemical oxidants were determined and the resistance profiles of the tested organisms established. Moreover, effects of the oxidants on cell membrane permeability and ATP production were compared among the CF and non-CF pathogens to assess whether the oxidant-induced damages correlate with bacterial viability.

## Methods

### Reagents and cultures

PsA, SA and BC were CF clinical isolates which were characterized by conventional microbiological methods including colony morphology, pigment production, Gram staining and standard biochemical tests [15]. KP (Strain 43816, serotype 2) was obtained from American Type Culture Collection (Manassas, VA). EC (Strain DH5α) was from Invitrogen (Carlsbad, CA). Percoll, 30% reagent-grade H_2_O_2_, and NaOCl (5% chlorine) were purchased from Fisher Scientific (Pittsburgh, PA). All cell and microbial culture media were purchased from Invitrogen.

### Microbial growth and storage

Luria-Bertani (LB) broth media (10 ml) were inoculated with PsA, SA, BC, KP or EC and cultured overnight at 37°C and 220 rpm. The following day, the cultures were streaked onto LB agar plates without antibiotics for colony isolation. New cultures were inoculated from single colonies of each organism and grown overnight at 37°C and 220 rpm. The pure cultures were cryogenically preserved by freezing a mixture of 0.5 ml of each culture with 0.5 ml of 30% glycerol in water at -80°C. Freshly streaked agar plate cultures for each organism were prepared from cryo stock bi-weekly.

### In vitro microbial killing with reagent H_2_O_2 _and HOCl

Bacterial cultures from isolation plates were grown overnight in LB broth media at 37°C with vigorous agitation at 230 rpm. On the day of experiments, the cultures were diluted 1:100 in LB broth media and subcultured to late-log phase. The subcultures were pelleted at 5000 × *g *and washed with Delbecco's Phosphate Buffered Saline (DPBS, pH 7.4, no Ca^2+ ^or Mg^2+^). The cell density was determined by the formula 1.0 OD_600 _= 1 × 10^9 ^cells/ml where OD_600 _is the optical density read at 600 nm in Beckman Coulter DU 640 spectrophotometer.

Oxidant-mediated killing by H_2_O_2 _and HOCl was carried out by modification of the methods described by McKenna and Davies [16]. For H_2_O_2_-mediated killing, microbes were suspended to 5 × 10^5 ^cells/ml in DPBS. Killing reactions were carried out in duplicate wells of 96-well microtiter plates containing 5 × 10^3 ^microbial cells in a final volume of 200 μl of DPBS containing various concentrations of H_2_O_2_. The mixtures were incubated at 37°C for 1 hour and were then transferred to ice to halt any additional growth. The samples were mixed by repeated pipetting just before plating 20 μl to LB agar plates. The plates were then incubated overnight at 37°C and the number of viable microbial cells for each H_2_O_2 _concentration was determined by colony forming unit (CFU) counting.

For HOCl-mediated killing, 5 × 10^8 ^bacterial cells were aliquotted, in duplicate, to 15 ml conical tubes at a final volume of 1 ml of DPBS containing various concentrations of HOCl as indicated. The tubes were incubated at 37°C for 1 hour with agitation and were then placed on ice. The samples were then passed through 25 gauge needles. Bacterial samples were then diluted 1:10^5 ^in DPBS. Fifty microliters of each diluted sample was plated to LB agar and cultured at 37°C. Microbial viability was assessed by CFU counting.

### Assessing HOCl- and H_2_O_2_-induced bacterial membrane permeability

Permeability of bacterial membranes after exposure of the organisms to reagent HOCl or H_2_O_2 _was measured using the LIVE/DEAD BacLight Bacterial Viability and Counting Kit (Molecular Probes, Carlsbad, CA). For HOCl-mediated membrane permeability studies, PsA, SA, KP, BC, and EC were grown in LB broth medium at 37°C overnight and subsequently subcultured (1:100) in fresh LB media until the culture reached late-log phase. The cells were then pelleted and washed with DPBS, quantified, and resuspended to 6.67 × 10^9 ^cells per milliliter. Cells (5 × 10^8^) were aliquotted to 15 ml conical tubes, and reagent NaOCl was added to the final concentrations indicated. The bacterial suspensions were incubated with the oxidant for 1 hour at 37°C and 220 rpm. The samples were placed on ice. Finally, the bacteria were pelleted in a table-top centrifuge at full speed for 2 minutes, and pellets were washed with ice-cold DPBS. The samples were stained according to manufacturer protocol with the vital dye Syto 9 as well as with propidium iodide (PI) which stains permeabilized cells. The percentages of fluorescently stained intact and permeable cells were assessed by flow cytometry, and the data were normalized to the oxidant-free controls. Controls for intact and permeable bacteria were produced by 1 hour incubation with either 0.85% NaCl or 70% ethanol, respectively, followed by washing and resuspension in 0.85% NaCl.

For H_2_O_2_-mediated membrane permeability studies, 1.25 × 10^6 ^cells were used per sample, each in a volume of 50 ml of DPBS to preserve the same cell density as was used in the above described CFU viability assay. Incubation times were the same as for the HOCl membrane permeability experiments. After incubation, the 50 ml samples were concentrated to 1 ml by centrifugation at 3000 × g for 15 minutes followed by washing, staining, and analysis as described above for HOCl assays. The controls were similarly set as well.

### Quantifying the effect of H_2_O_2 _and HOCl on bacterial ATP production

The indicated organisms were exposed to H_2_O_2 _or HOCl as indicated above in the membrane permeability studies. ATP production was quantified following oxidant exposure using the BacTiter-Glo Microbial Cell Viability Assay from Promega according to manufacturer protocol. 5 × 10^6 ^cells were used in each assay sample to yield a signal-to-noise ratio of approximately 10^4^-10^5^:1. ATP-specific luminescence was measured using a BioTek (Winooski, VT) Synergy HT microplate reader, and ATP concentration was determined by fitting the luminescence values to a standard curve generated using 10-fold dilutions of Na-ATP from 1 μM to 10 pM. Data are represented as percent ATP recovery relative to oxidant-free controls.

### Statistical analysis

Two-way ANOVA with replication was used when analyzing organism viability. Differences in the single parameter of membrane integrity or ATP level were analyzed by One-way ANOVA. Linear regression was performed for correlating membrane permeability and ATP production with bacterial CFU viability.

## Results

### Oxidant resistance of CF and non-CF pathogens to H_2_O_2 _and HOCl

**Figure 1:**
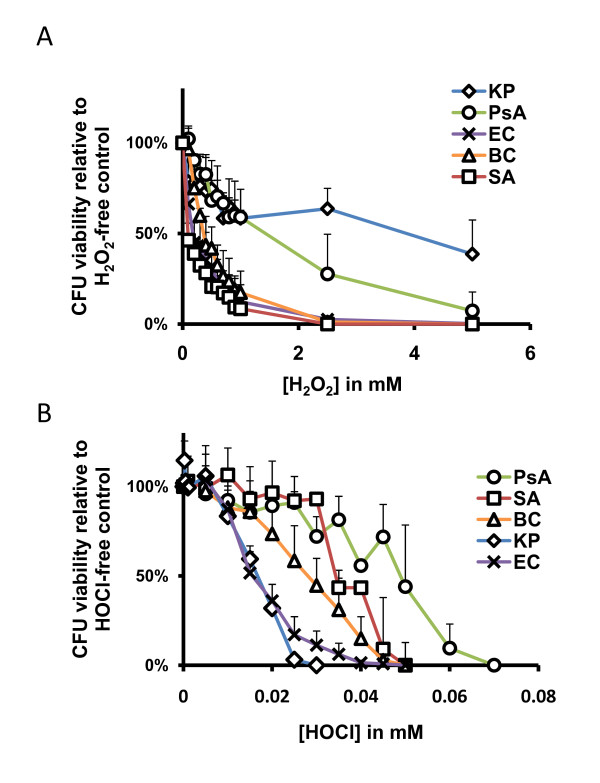
**Bacterial killing by reagent H**_**2**_**O**_**2 **_**and HOCl *in vitro***. Microbes were exposed to various concentrations of H_2_O_2 _or HOCl, as indicated, for 1 hour at 37°C. At the end of the exposure, the samples were plated to LB agar plates for overnight culture. Bacterial killing by oxidants was measured as percent of viable bacteria relative to the number of colonies from the oxidant-free controls. **A**) Organisms indicated were exposed to 0 mM to 5.0 mM H_2_O_2 _or (**B**) 0 mM to 0.1 mM HOCl. PsA = *P. aeruginosa*, SA = *S. aureus*, BC = *B. cepacia*, KP = *K. pneumoniae*, and EC = DH5α-*E. coli*. Error bars represent standard deviation of at least *n *= 3 experiments.

We exposed PsA, SA, KP, BC, and EC to reagent-grade H_2_O_2 _or HOCl, *in vitro*, to compare their intrinsic susceptibility or resistance as described in Materials and Methods. The results (Figure 1A) demonstrated that KP and PsA were the most resistant organisms to H_2_O_2_. Unexpectedly, KP, a non-CF pathogen, showed almost an equal, if not greater, resistance to H_2_O_2 _than PsA by two-way ANOVA test (p = 0.79; Figure 1A and Table 1). Both PsA and KP were vastly more resistant to H_2_O_2 _than any of the other organisms tested (*p *< 0.0001 for all comparisons). BC, SA, and EC were the most susceptible to H_2_O_2 _with approximately 90% eradication at approximately 1 mM of the oxidant. Statistically, the profile of greatest to least H_2_O_2_-resistant organisms is as follows: KP > PsA > BC > EC > SA.

**Table 1:** Comparisons of H_2_O_2 _*in vitro *killing of various species of bacteria (P-value from two-way ANOVA with replication)

	PsA	SA	BC	KP	EC
**PsA**	-	<0.0001	<0.0001	0.79	<0.0001

**SA**	<0.0001	-	<0.0001	<0.0001	0.0006

**BC**	<0.0001	<0.0001	-	<0.0001	0.0002

**KP**	0.79	<0.0001	<0.0001	-	<0.0001

**EC**	<0.0001	0.0006	0.0002	<0.0001	-

When exposed to HOCl, the organisms tested displayed a different profile of oxidant-mediated killing. Other than the fact that HOCl is vastly more microbicidal to all the organisms tested at lower concentrations than H_2_O_2_, the most noticeable difference was the sharp decline in viability of KP with increasing HOCl concentration (Figure 1B). Where we previously observed strong resistance of KP to H_2_O_2_, here it appeared to be among the most susceptible to HOCl assault. PsA and SA emerged as the most resistant organisms to HOCl-mediated killing, and the difference between the two organisms was not statistically significant (*p *= 0.39; Table 2). However, the killing curves of PsA and SA did terminate at slightly different values; that is, complete abolition of CFU formation occurred at 0.05 mM HOCl for SA while PsA was not completely eradicated until the HOCl concentration reached 0.07 mM. Both PsA and SA killing curves were significantly different from that of BC (*p *< 0.0001), and BC survived HOCl-mediated assault at significantly higher concentrations than did KP or EC (*p *= 0.006 and *p *< 0.0001, respectively). Under these conditions, the profile of greatest to least HOCl-resistant organisms is as follows: PsA > SA > BC > EC > KP.

**Table 2:** Comparisons of HOCl *in vitro *killing of various species of bacteria (P-value from two-way ANOVA with replication)

	PsA	SA	BC	KP	EC
**PsA**	-	0.39	<0.0001	0.0007	<0.0001

**SA**	0.39	-	<0.0001	0.004	<0.0001

**BC**	<0.0001	<0.0001	-	0.006	<0.0001

**KP**	0.0007	0.004	0.006	-	0.02

**EC**	<0.0001	<0.0001	<0.0001	0.02	-

Based on the above oxidant-resistance data, we recognized that the HOCl bacterial killing profile remarkably fit the infection profile observed in CF patients clinically. Among the CF and non-CF pathogens tested, PsA was the strongest organism resistant to both oxidants.

### Oxidant-induced membrane injury of CF and non-CF pathogens

The bacterial membrane is the first contact point for oxidants to act on these cells. To examine effects of the oxidants on bacterial membrane integrity, we measured the cell permeability before and after oxidant exposure. The uptake of fluorescent Syto9, a cell vital dye, and propidium iodide (PI), a permeable cell dye, were analyzed by flow cytometry. The percent of cells with intact cytoplasmic membranes were compared and normalized to the percent of bacteria with the intact membranes in the oxidant-free controls.

**Figure 2:**
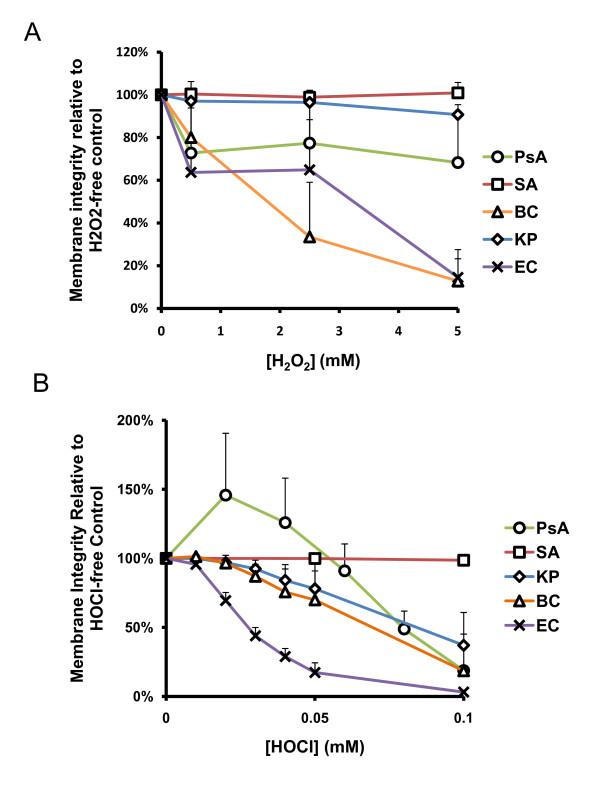
**H**_**2**_**O**_**2 **_**and HOCl-induced membrane permeability**. Bacteria were exposed to reagent **A) **H_2_O_2 _or **B) **HOCl as indicated, and the effect of the oxidant on membrane integrity was measured by the BacLight Bacterial Viability and Counting Kit (Molecular Probes). Membrane integrity of PsA, SA, and KP were not significantly affected by H_2_O_2 _up to 5 mM by single-factor ANOVA analyses. All organisms tested demonstrated HOCl dose-dependent membrane permeability except SA which remained unaffected up to 0.1 mM. Error bars represent standard deviations of at least n = 3 experiments.

**Figure 3:**
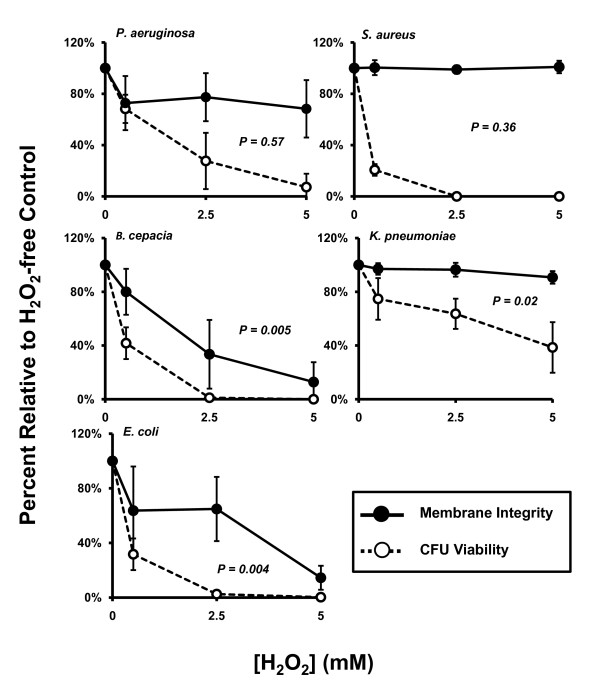
**Correlating H**_**2**_**O**_**2**_**-mediated membrane permeabilization and CFU viability**. For BC, KP, and EC, loss of membrane integrity correlated statistically with decline in CFU viability while these two parameters were statistically independent of each other for PsA and SA. Solid circles and lines: membrane integrity. Open circles and dotted lines: bacterial viability. Both parameters were expressed as percent relative to oxidant-free controls. *P*-values represent linear regression of the raw data values from membrane permeability versus bacterial viability. Values less than 0.05 were considered significant and denote correlation between the parameters; values greater than 0.05 indicate independence of the parameters. Error bars represent standard deviation of at least n = 3 experiments.

The membrane integrity of PsA, SA, and KP were not significantly affected by H_2_O_2 _up to 5 mM, the maximum concentration measured herein, as compared to each corresponding buffer controls. Single factor (One-way) ANOVA analyses revealed a *p *value of 0.22, 0.94 or 0.12 for PsA, SA or KP, respectively (Figure 2A). BC and EC displayed increasing percentages of permeable cells after exposure to H_2_O_2 _from 0 mM to 5 mM (*p *= 0.0008 and 0.006, respectively) with 50% permeability occurring at approximately 2.5 mM for each. To relate the membrane damage to cell viability, we performed linear regression test for each organism. As shown (Figure 3), the percent of PsA and SA with intact membranes after exposure to a range of H_2_O_2 _concentrations relative to the oxidant-free control was statistically independent from the measure of CFU viability at the same peroxide concentrations (*p *= 0.57 and 0.36, respectively), suggesting the two phenomena were not related. Declining CFU viability from exposure to increased peroxide concentration did correlate statistically with the loss of membrane integrity after exposure of BC and EC to H_2_O_2 _(*p *= 0.005 and 0.004, respectively). Though membrane integrity of KP was statistically unaffected by H_2_O_2 _exposure while CFU viability did significantly decline with increasing H_2_O_2 _concentration, the two parameters are not statistically independent of each other (*p *= 0.02).

**Figure 4:**
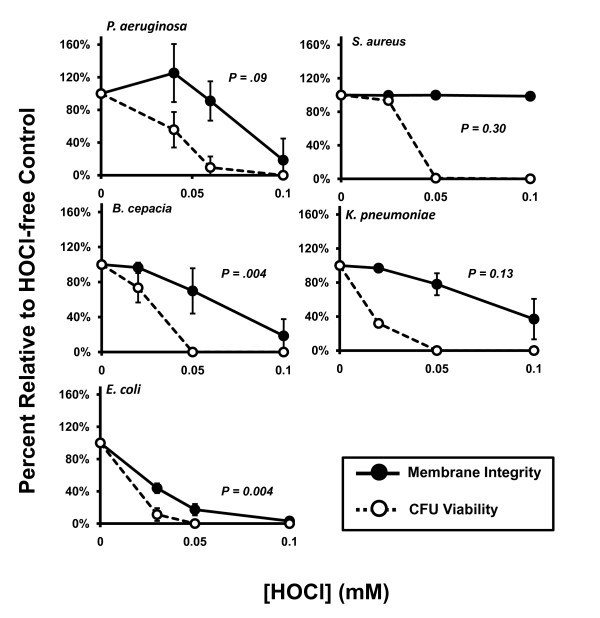
**Correlating HOCl-induced membrane permeability and CFU viability**. Bacteria were exposed to reagent HOCl *in vitro *to determine the effect of the oxidant on membrane integrity as measured by the BacLight Bacterial Viability and Counting Kit (Molecular Probes). Concentrations of HOCl used were based on the amounts necessary to eradicate CFU viability as assessed in the previous experiments. In general, bacterial membranes remain intact at concentrations beyond that required to inhibit CFU formation and kill the organism. Under these conditions, PsA, SA, and KP were killed at statistically lower concentrations of HOCl than were required to produce the same degree of membrane permeabilization. Membrane permeabilization by HOCl in BC and EC correlated with loss of CFU viability. Solid circles and lines: membrane integrity. Open circles and dotted lines: bacterial viability. Both parameters were expressed as percent relative to oxidant-free controls. *P*-values represent linear regression of the raw data values from membrane permeability versus CFU viability. Values less than 0.05 were considered significant and denote correlation among the parameters; values greater than 0.05 indicate independence of the parameters. Error bars represent standard deviation of at least n = 3 experiments.

Membrane integrity of PsA, BC, KP, and EC was affected significantly by exposure to up to 0.1 mM HOCl, in a dose-dependent manner, as compared to each corresponding buffer controls (*p *= 0.007, 0.003, 0.002, and <0.0001, respectively, by One-way ANOVA test; Figure 2B), while SA membrane integrity was unaffected by these concentrations. Furthermore, linear regression tests, shown in Figure 4, revealed that CFU viability was abolished at lower concentrations than those required to produce the same degree of membrane permeabilization in PsA, SA, and KP; that is, no correlation was detected between these two parameters for each organism (*p *= 0.09, 0.30, and 0.13, respectively). BC and EC demonstrated HOCl-induced membrane permeabilization that correlated significantly with CFU viability of these organisms after oxidant exposure (*p *= 0.004, and 0.004, respectively). EC membrane integrity was most affected by HOCl exposure with 50% permeabilization occurring between 0 mM and 0.03 mM while all other organisms with affected membrane integrity lost 50% integrity between 0.05 mM and 0.1 mM.

### Effect of oxidants on bacterial ATP production

**Figure 5:**
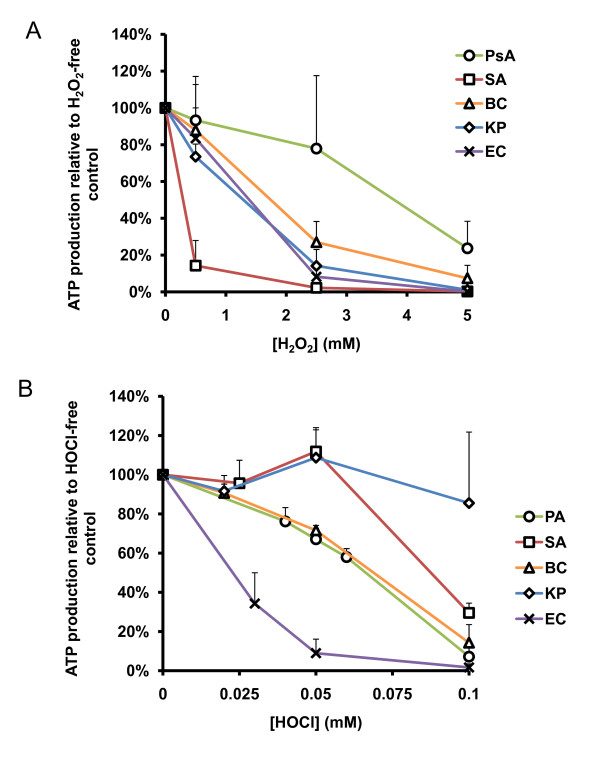
**H**_**2**_**O**_**2**_**- and HOCl-induced ATP changes in bacterial pathogens**. Bacteria were exposed to reagent H_2_O_2 _or HOCl, *in vitro*, to determine the effect of the oxidant on ATP production as measured by the BacTiter-Glo Microbial Cell Viability Assay (Promega). Concentrations of oxidants used were based on the amounts necessary to eradicate CFU viability as assessed in the previous experiments. **A**) All organisms displayed significant reduction in ATP production (One-way ANOVA) in an H_2_O_2 _dose-dependent manner up to 5 mM. **B**) ATP production by KP was statistically unaffected by HOCl exposure up to 0.1 mM according to one-way ANOVA (*p *= 0.53) while all other organisms tested displayed significant HOCl dose-dependent reduction in ATP production in this concentration range. Error bars represent standard deviation of at least n = 3 experiments.

**Figure 6:**
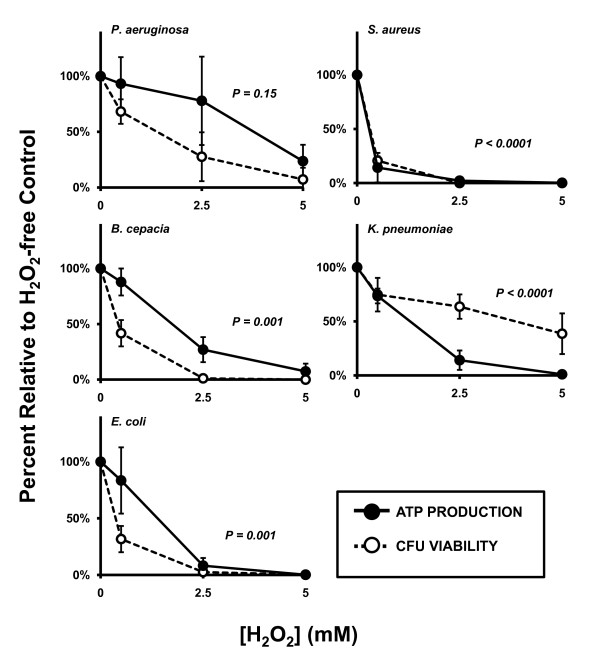
**Correlating H**_**2**_**O**_**2**_**-induced loss of ATP production with bacterial viability**. H_2_O_2_-induced disruption of ATP production correlated statistically with abolishment of CFU viability for all organisms tested except PsA (*p *= 0.15) at concentrations up to 5 mM. Though the decline of ATP production in PsA for this oxidant was statistically significant in this range, the percent change remains independent of the percent reduction in CFU viability. Solid circles and lines: ATP recovery after oxidant exposure. Open circles and dotted lines: CFU viability. Both parameters are measured as percent relative to oxidant-free controls. *P*-values represent linear regression of the raw data values from percent ATP recovery versus CFU viability. Values less than 0.05 were considered significant and denote correlation between the parameters; values greater than 0.05 indicate independence of the parameters. Error bars represent standard deviation of at least n = 3 experiments.

Energy supply is another house-keeping factor vital to bacterial viability. Because the F_1_F_0 _ATP synthase is a cell membrane-bound protein which is exposed to outside, oxidants applied may preferentially target the energy production system. A previous publication has reported that ATP production is a major target of oxidants [17]. Here, we treated the CF and non-CF pathogens with H_2_O_2 _from 0 mM to 5.0 mM or with HOCl from 0 mM to 0.1 mM for 1 hour at 37°C. After oxidant exposure, the bacteria were analyzed for cellular ATP levels. All organisms tested displayed significant reduction in ATP content with increasing doses of H_2_O_2 _by One-way ANOVA analyses (PsA, *p *= 0.02; SA, *p *< 0.0001; BC, *p *< 0.0001; KP, *p *< 0.0001 and EC, *p *< 0.0001; Figure 5A). This reduction correlated statistically with CFU viability under the same conditions for all organisms except PsA which failed to reach statistical correlation by linear regression analysis (Figure 6) (SA: *p *< 0.0001; BC: *p *= 0.001; KP: *p *< 0.0001; EC: *p *= 0.001 and PsA: *p *= 0.15). Interestingly, the relative H_2_O_2 _dose-dependent decline in ATP content in KP was more dramatic than the loss of CFU viability under the same conditions.

**Figure 7:**
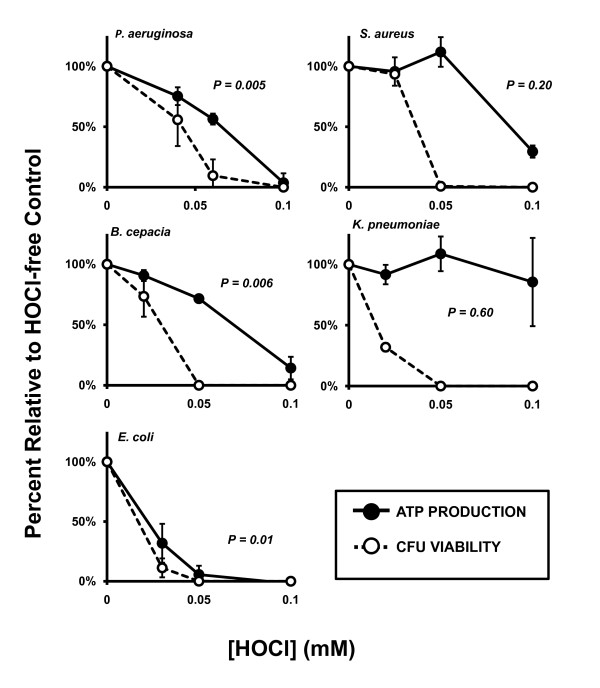
**Correlating HOCl-induced ATP changes with bacterial viability**. ATP production is affected by HOCl exposure and correlates statistically with CFU viability in PsA, BC, and EC (p = 0.005, 0.006, and 0.01, respectively); however, SA and KP lose CFU viability after exposure to lower concentrations of HOCl than are required to abolish ATP production during the assay time. Solid circles and lines: ATP recovery after oxidant exposure. Open circles and dotted lines: CFU viability. Both parameters are measured as percent relative to oxidant-free controls. *P*-values represent linear regression of the raw data values from percent ATP recovery versus CFU viability. Values less than 0.05 were considered significant and denote correlation among the parameters; values greater than 0.05 indicate independence of the parameters. Error bars represent standard deviation of at least n = 3 experiments.

ATP production was dose-dependently abolished in PsA, SA, BC, and EC while KP remained statistically unaffected even at HOCl doses up to 0.1 mM (PsA, *p *< 0.0001; SA, *p *< 0.0001; BC, *p *< 0.0001; EC, *p *< 0.0001 and KP, *p *= 0.53; Figure 5B). The decline in ATP production correlated with HOCl-induced loss of CFU viability in PsA, BC, and EC (*p *= 0.005, 0.006, and 0.01, respectively, Figure 7) but was independent of diminished CFU viability in SA and KP (*p *= 0.20 and 0.60, respectively).

## Discussion

In this report, we used a cell-free system with reagent H_2_O_2 _and HOCl to examine the intrinsic resistance of prevalent CF and Non-CF respiratory pathogens to the oxidants. We found that the *in vitro *HOCl-resistance profile (PsA > SA > BC > EC > KP) best fits the infection profile observed clinically in CF lungs; that is, the most HOCl-resistant bacteria such as PsA and SA are the most frequent pathogens in CF patients. This finding implies that differential HOCl resistance across microbial species may allow for persistence of some infections over others by subversion of the host innate immunity and supports our previous finding that CF neutrophils with a compromised HOCl production may not be able to clear the most resistant organisms effectively [12,13]. From a microbiological point of view, PsA and SA, the relatively more resistant strains to HOCl, would be more likely to survive and be selected for, if the host neutrophils were deficient in their ability to make HOCl. Burns and coworkers did a longitudinal study on young children with CF and found that 97% of the children are colonized with PsA [18]. The early isolates tend to be nonmucoid and antibiotic-sensitive. However, if the initial infection is not effectively eradicated by the host defense, which could happen, for example, if HOCl or other oxidant production was suboptimal, then the bacteria which escape the initial host defenses will grow and spread within the lung, establishing a long-term chronic colonization. Subsequently, environmental pressure in the lung such as antibiotic application selects for the mucoid PsA phenotype. Increased PsA density in the lower respiratory tract and development of antibiotic-resistant mucoid biofilms causes chronic airway inflammation and deteriorating lung function [19-22]. SA has long been recognized to be among the first organisms to colonize the airways of CF patients [23]. Colonization with SA occurs within the first few months of life, and persistent variants of this organism may arise due to a selective pressure from long-term antibiotic treatment in CF patients [24]. However, SA infection does not usually persist or progress to chronic disease. We would like to point out that our current study only tested bacteria in log-phase growth. Such an experimental design was intended to study the nonmucoid form which is assumed by the bacteria during the early CF infections. It is important to recognize that only after initial bacterial colonization is established, can chronic persistent infections ensue in CF lungs.

Neutrophils are highly specialized for bacterial killing especially in the case of extracellular infections. The cells employ at least two microbicidal mechanisms to execute this function: one is oxidant-mediated and another is non-oxidant-mediated. *Pseudomonas *bacteria possess tough polysaccharide capsules, which are resistant to nonoxidant killing mechanisms, such as protease and hydrolase digestion [25]. This feature determines the requirement of oxidant-killing mechanisms for complete eradication. The importance of neutrophils in defending *Pseudomonas *infection is reflected by significant increase in infection rate in neutropenic patients [4]. Winterbourn and colleagues modeled the reactions of oxidant production in neutrophil phagosomes. They calculated that superoxide is produced at a rate of ~312 mM/min and HOCl 134 mM per minute [10]. In this current study, the maximal concentration of H_2_O_2 _used was 5 mM and HOCl 0.07 mM. A recent report documented that bleaching of GFP expressed in SA is seen at concentrations of 0.05-0.1 mM HOCl which correlated well with killing of SA by this oxidant [26], suggesting that similar concentrations of HOCl were likely achieved *in vivo*. The mathematical model proposed by Winterbourn and colleagues predicts that such levels can be reached within seconds after activation of the NADPH oxidase [10]. Thus, we believe that the selected concentrations of H_2_O_2 _and HOCl in our studies are well within the scope of the achievable oxidant levels in neutrophils.

Precise mechanisms of oxidant-mediated bacterial killing are not fully defined. Early studies using EC as a model organism indicated a correlation between EC envelope permeabilization and bacterial inactivation by HOCl; however, only low-molecular weight compounds became freely permeable while the cell maintained its barrier function to proteins [27]. Albrich *et al*. (1986) tested the small-molecule permeability theory in EC by measuring the transport of H^+ ^ion and glycerol and reported that the intercellular movements of these molecules were only marginally affected [28]. Their conclusion was that HOCI inactivation of bacteria does not occur by loss of membrane structural integrity, which contradicts the previous report. In the current study, we demonstrated that membrane integrity is affected by H_2_O_2 _and HOCl, but the effect is organism-specific (Figures 2 and 3). Statistically, permeability of BC and EC caused by H_2_O_2 _and HOCl did correlate with loss of viability while permeability of KP with only H_2_O_2 _exposure correlated with loss of viability. It is notable that permeability and CFU viability were statistically independent of each other for PsA and SA, the two most prevalent CF pathogens, in both H_2_O_2 _and HOCl exposures.

EC and PsA have been shown to recover from reduced adenylate energy charge, when subsequently supplied with nutrients which facilitate ATP hydrolase activity of the F_1_F_0 _complex of the bacterial ATP synthase [29]. After treatment with bactericidal doses of HOCl, however, adenylate energy charge is unrecoverable and further ATP production is abolished [17]. These findings suggest that a potent oxidant-induced killing mechanism may cause destruction of ATP production by specific oxidation of the F_1_F_0 _ATP synthase [30]. We measured the ATP concentration in bacterial suspensions after exposure to H_2_O_2 _and HOCl exposure and compared the degree of ATP reduction to the degree of loss of CFU viability at various oxidant concentrations (Figure 4). Of the organisms tested, all except PsA demonstrated significant decline in ATP production which correlated with loss of CFU viability; ATP production in PsA declined significantly up to 5 mM but did not correlated with decline in CFU viability. These data present evidence that H_2_O_2 _affects ATP production in bacteria suggesting that there are H_2_O_2_-sensitive sites in the bacterial ATP production machinery or that H_2_O_2 _assault disrupts pathways of energy production.

The profile of abolished ATP production with HOCl treatment was different from that of H_2_O_2 _in that HOCl-induced loss of ATP production correlated significantly with the loss of CFU viability in PsA, BC, and EC, while these two parameters were statistically independent in SA and KP (Figure 5). Interestingly, ATP production in KP was unaffected by HOCl concentrations up to 0.1 mM, a dose exceeding that required for complete eradication of the entire samples at the cellular densities used herein. Given the results obtained in SA and KP, it can be inferred that loss of CFU viability is not completely dependent on disruption of ATP production. In light of these results, further studies are required to elucidate the specific mechanisms of oxidant-induced bactericidal activity against different bacterial species.

## Conclusions

We have demonstrated that the HOCl-resistance profile of microorganisms relates to its clinical pathogenicity in CF lung disease. Therefore, defective oxidant-mediated phagocytic host defense in CF may predispose the patient to chronic infections, especially those caused by PsA. Furthermore, oxidants affect bacterial membrane permeability and ATP energy production. But the effects are organism-specific, indicating that varied survival advantages exist among the bacteria when they are phagocytosed and encounter phagocyte-produced oxidants.

## Authors' contributions

RWB performed experiments, data analyses and manuscript writing; RGP provided technical assistance and experimental design; EML contributed to statistical analysis; GW did experimental design, data interpretation and manuscript writing. All authors read and approved the final manuscript.
